# Report of the Board of Health of the City of Philadelphia for 1862

**Published:** 1863

**Authors:** 


REPORT OF THE BOARD OF HEALTH OF THE CITY AND PORT OF PHILADELPHIA FOR 1862. BY JAMES A. MCCREA, M.D., AND WILLIAM READ, ESQ.

This is a well-printed pamphlet of about fifty pages, containing much statistical information of value concerning the Births, Marriages, Deaths, and Diseases of Philadelphia during the year 1862.

